# Telomere Length Regulation

**DOI:** 10.3389/fonc.2022.943622

**Published:** 2022-07-04

**Authors:** Peter Lansdorp

**Affiliations:** ^1^ Terry Fox Laboratory, British Columbia (BC) Cancer Agency, Vancouver, BC, Canada; ^2^ Department of Medical Genetics, University of British Columbia, Vancouver, BC, Canada

**Keywords:** telomerase, telomere length measurements, telomere replication, telomere length regulation, development, quadruplex DNA, tumor suppression, lifespan

## Abstract

The number of (TTAGGG)_n_ repeats at the ends of chromosomes is highly variable between individual chromosomes, between different cells and between species. Progressive loss of telomere repeats limits the proliferation of pre-malignant human cells but also contributes to aging by inducing apoptosis and senescence in normal cells. Despite enormous progress in understanding distinct pathways that result in loss and gain of telomeric DNA in different cell types, many questions remain. Further studies are needed to delineate the role of damage to telomeric DNA, replication errors, chromatin structure, liquid-liquid phase transition, telomeric transcripts (TERRA) and secondary DNA structures such as guanine quadruplex structures, R-loops and T-loops in inducing gains and losses of telomere repeats in different cell types. Limitations of current telomere length measurements techniques and differences in telomere biology between species and different cell types complicate generalizations about the role of telomeres in aging and cancer. Here some of the factors regulating the telomere length in embryonic and adult cells in mammals are discussed from a mechanistic and evolutionary perspective.

## Introduction

Ever since it was observed that telomere repeats are lost with each DNA replication cycle *in vitro* (1) as well as with age in human tissues (2, 3) and that such losses are mechanistically linked to replicative senescence (4), the role of telomeres and telomerase in aging and cancer has been the subject of intensive research efforts (reviewed in (5–8)). As a result, the amount of relevant information can no longer be effectively communicated in a single review, posing a major challenge for researchers entering the field. This dilemma is by no means unique to the telomere field and this perspective does not pretend to solve it. Instead, it is hoped that a discussion of selected studies and observations will inspire discussion, insights and new experiments. For this purpose, a general discussion of the mechanisms involved in the elongation and the shortening of telomeres is followed by a discussion of factors and pathways that are implicated in regulating the number of telomere repeats at chromosome ends in embryonic and adult cells.

## Telomere Length Regulation: “The Landscape”

That complete replication of linear chromosomes could represent a problem was already proposed in the early 1970s (9, 10) and the “end-replication problem” is a now a well-recognized cause of telomere attrition (reviewed in (11). In most organisms with linear chromosomes the solution to the “end-replication problem” is extension of the 3’ end of DNA template strands by means of telomerase, a specialised reverse transcriptase (12, 13). However, telomerase is not the only solution to the “end replication problem”. For example, *Drosophila melanogaster* uses a transposition mechanism to replenish the DNA lost with each DNA replication cycle [reviewed in (14)].

Unfortunately, understanding the Yin and Yang at the telomere: the end replication problem and its primary solution, telomerase, only scratches the surface of why all chromosomes end up with a given number of telomeric repeats at any given point in time. While much progress has been made in the characterization of the molecules and processes involved in the loss and gain of telomere repeats at chromosome ends, many questions remain unsolved. Further work is needed to better understand the role of telomerase and its interaction with chromosome ends, the role of telomere chromatin (reviewed in (15) and the “shelterin” proteins that bind to telomeric DNA (reviewed in (16) in telomere function in different cell types during development and adult tissues. The “shelterin” complex is a set of related complexes that not only differ between cells but also function differently along the telomere at different stages of the cell cycle (reviewed in (7). Other areas that require further study are how cells respond to the DNA damage signals derived from critically short telomeres and how the composition of “shelterin” proteins is modulated by telomeric TERRA transcripts (reviewed in (17), R-loops and guanine quadruplex (G4) structures (reviewed in (18, 19).

Apart from differences between cell types, a complicating factor is that the regulation of telomerase and telomere length in various cell types is markedly different between short- and long-lived animals (reviewed in (5). Low levels of telomerase limit the replicative potential of somatic cells in long-lived animals including humans but not in relatively short-lived animals such as laboratory mice. Most likely, replication-dependent telomere erosion in somatic cells protects long-lived animals against tumors early in life be it at the expense of tissue regeneration and immune responses late in life. This idea, captured in the “telomere erosion in disposable soma (TEDS)” theory of human aging (5), provides the framework for the following discussion of telomere length regulation in human cells.

## Loss of Telomeric DNA

Next to the loss of telomeric DNA *via* the “end-replication problem”, telomeric DNA is also lost *via* the obligatory processing of chromosome ends following leading strand DNA replication (20). The resulting blunt ends must be processed in order to create a single strand 3’ overhang that is presumed to be present and required at every chromosome end. Apart from inevitable losses of telomeric DNA with each replication cycle, telomeric DNA can also be lost by other, less predictable mechanisms. Sporadic problems can arise following various types of damage to telomeric DNA but also when single stranded G-rich telomeric DNA is allowed to form secondary structures that interfere with replication or repair. In principle, all processes that disrupt duplex telomeric DNA can generate single stranded DNA which in turn can form secondary DNA structures known as guanine quadruples (G4) structures (21). Next to G4 structures, single stranded G-rich DNA, folded back into duplex telomeric DNA called T-loops, and RNA transcripts of telomeric DNA called TERRA, associated with telomeric DNA in structures called R-loops, can also cause stalling of the replication fork (22–24). In view of the variable length of TERRA and single stranded G-rich DNA telomere repeats, a variety of G4 structures could form at telomeres including stable hybrid G4 structures containing both RNA and DNA (25).

Unlike elsewhere in the genome, where a stalled replication fork can be rescued by a fork coming from the opposite direction, rescue by incoming forks is not expected at telomeres unless replication is initiated from origins within the telomere upon replication stress (26). Progression of the replication fork at telomeres can also be hampered by other types of DNA lesions including intra-strand crosslinks as well as tightly DNA-bound proteins in telomeric heterochromatin (11). Stalled replication forks can lead to fork collapse and breaks in telomeric DNA. When such breaks are not repaired by telomerase or recombination (27), large tracts of telomere repeats are lost.

**Figure 1:**
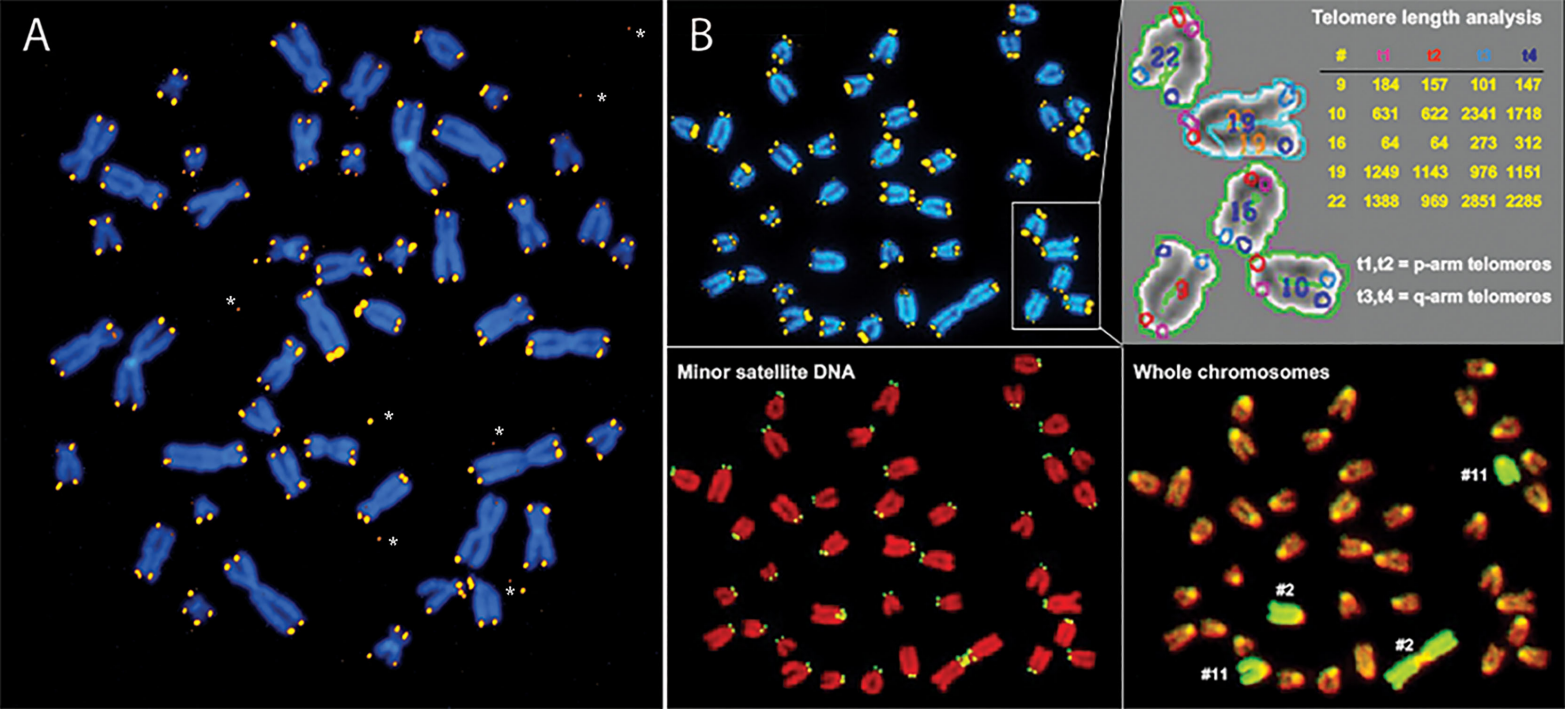
Q-FISH shows extreme variability in the length of telomere repeats in human **(A)** and mouse **(B)** chromosomes. **(A)** Human metaphase chromosomes stained with DAPI (blue) from a cultured fibroblast following hybridization with Cy3 labeled (CCCTAA)_3_ PNA (yellow). Note the large variation in telomere fluorescence intensity at individual chromosome ends, discrepancies in telomere fluorescence between sister chromatids and occasional telomere fluorescence spots outside chromosomes*. **(B)** Mouse metaphase chromosomes from a cultured skin fibroblast hybridized sequentially with a mixture of different fluorescent probes: first Cy3 labeled (CCCTAA)_3_ PNA (**B**, top left panel) and FITC labeled CGGCATTGTAGAACAGTG PNA specific for mouse minor satellite sequences (**B**, bottom left panel) followed by staining of DNA with DAPI, image acquisition and hybridization with FITC labeled chromosome paint probe specific for respectively chr2 and chr11 (**B**, bottom right panel). Telomere length was analyzed using the TFL-Telo software (**B**, top right panel) (28). Note the very short telomeres on the short arm of chr “16” and the very long telomeres on the long arm of chr “22” (arbitrary chromosome numbers and fluorescence intensity values). For details see (29, 30).

Observations of telomeric DNA at individual chromosome ends in human and murine cells using quantitative fluorescence *in situ* hybridization (Q-FISH) documented heterogeneous telomere length distributions as well as sporadic loss of telomeric DNA (**Figure 1**) (5, 30–32). Importantly, in cells with low or undetectable telomerase levels such as human fibroblasts, the biological consequences of sporadic losses of telomeric DNA add to the inevitable loss of telomere repeats with each replication cycle to accelerate the replicative senescence or apoptosis of cells.

## Solving Additional Problems That Arise During Telomere Replication

Most problems at telomeres probably arise or become apparent during DNA replication. Apart from the inevitable problems during replication discussed above several additional problems have been identified (reviewed in (11)). Lagging strand replication of telomeric DNA involves either the regular lagging strand DNA replication complex or a replication complex that is more specific to telomeres involving CTC1, STN1 and TEN1, a set of proteins known as the CST complex (7). The CST complex is similar but different from RPA, the protein complex that typically binds single strand DNA. The CST complex is required for “fill-in” synthesis of C-rich DNA from G-rich templates extended or produced by telomerase. Where or when lagging strand replication switches from RPA to CST during replication of telomeric DNA in the absence of telomerase is not known. CST protein levels could be important to suppress the formation of G4 structures from single stranded G-rich DNA at telomeres produced by telomerase or exposed during replication, transcription or recombination. The POT1 protein is another protein that binds to single stranded G-rich DNA to suppress the formation of G4 structures (33) and the full extent of its roles in telomere biology remains to be clarified (34, 35). In general, more studies are needed to better understand the role of DNA damage response pathways in telomere function. Such studies should elucidate the balance between proteins that suppress the formation of G4 DNA at telomeres and helicases such as RTEL1, BLM and FANCJ that are capable of unwinding G4 structures (36). Differences between cell types and the stochastic nature of G4 DNA formation provide significant challenges for such studies.

## Multiple Molecular Exchanges at Telomeres

**Figure 2:**
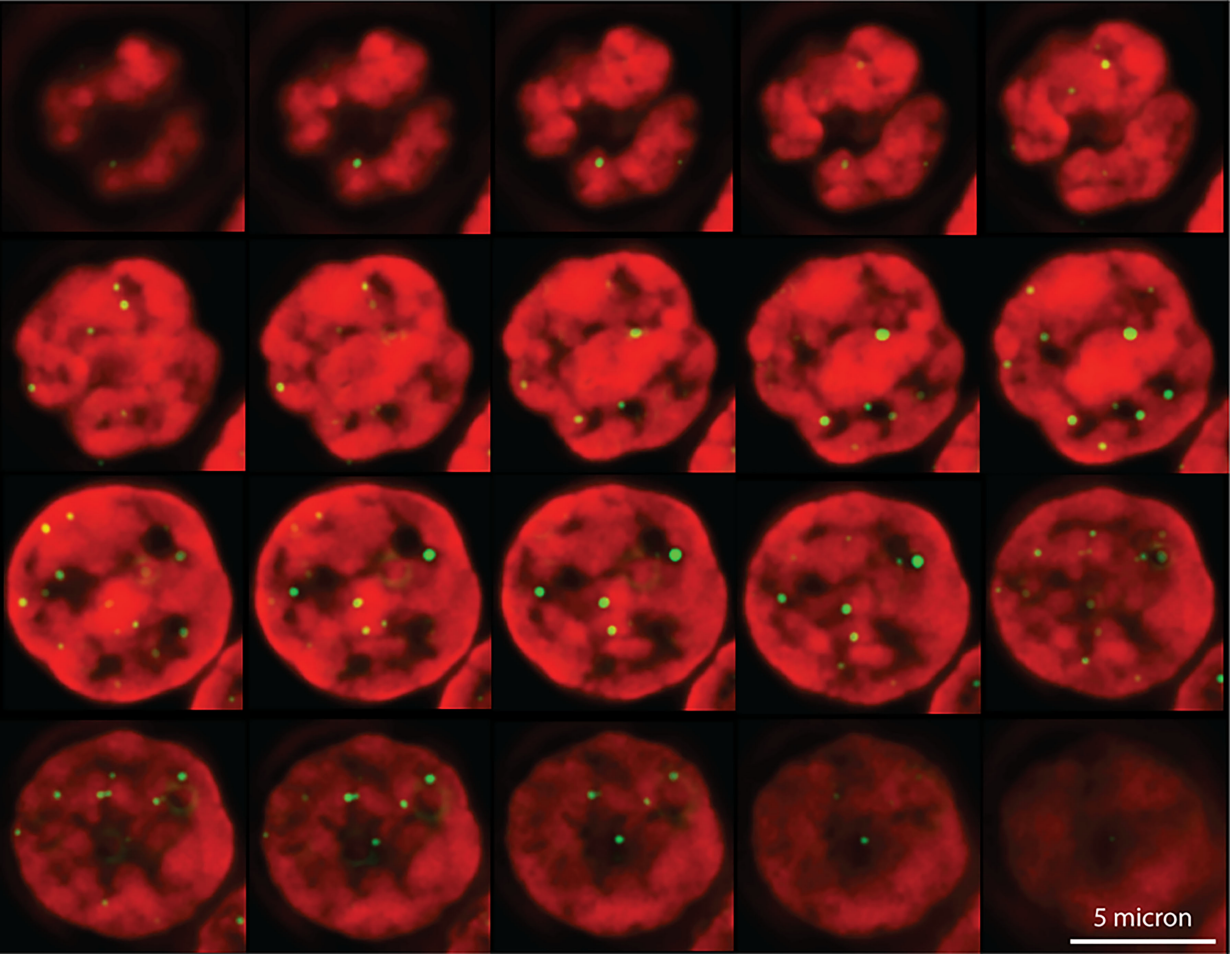
Telomeres in a human lymphocyte are not randomly distributed in the nucleus. Shown are optical sections through the interphase nucleus of a human T lymphocyte following formaldehyde fixation and fluorescence *in situ* hybridization with fluorescently labeled (CCCTAA)_3_ PNA (shown in yellow/green). DNA is counterstained with DAPI (shown in red). A stack of images, acquired at separate focal planes, was processed using deconvolution microscopy (40). Note that telomeres appear to cluster at the interface between DNA bright areas, presumably reflecting heterochromatin and DNA weakly stained areas, presumably representing euchromatin (Chavez and Lansdorp unpublished observations).

**Figure 3:**
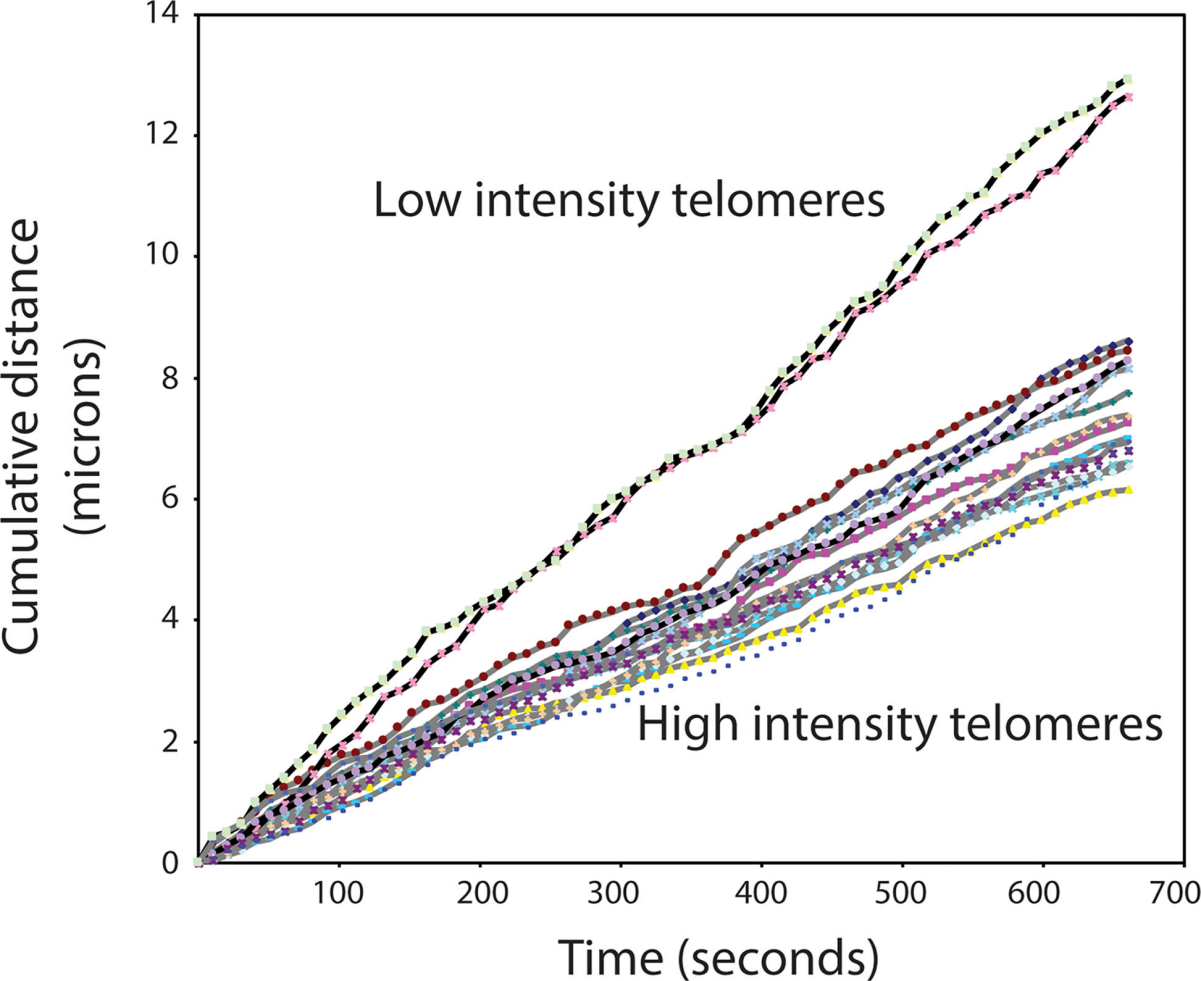
High mobility of very short telomeres in cultured mouse embryonic stem cells. Viable cells, tagged with Venus-TRF1, were imaged at a fixed position over 10 minutes. The position of individual fluorescent telomere spots was recorded every 10 seconds. Two categories of telomere spots were observed: low intensity spots and high intensity spots. The recorded position of each spot at each time interval was used to calculate the travel distance of individual telomeres. See **Supplementary Information** and **Supplementary Movie 3** for details.

The switch between a protected telomere end and a chromosome end that signals DNA damage has many levels and components. Many of these components themselves have switch-like characteristics in that two alternate states can be present or selected. For example, it is possible that telomere damage signaling involves a liquid-liquid phase separation in the nucleus between heterochromatin and euchromatin (reviewed in (37–39). Both the position of telomeres in the nucleus and studies of telomere mobility provide some support for this possibility. Telomeres in human lymphocytes are not randomly distributed in the nucleus but appear positioned at the interface between euchromatin and heterochromatin (**Figure 2**). Interestingly, when the movement of telomeres in cultured mouse embryonic stem cells was studied using telomere binding protein tagged with green fluorescent protein (Venus-TRF1), it was observed that the faintest fluorescent spots showed the highest mobility. These findings are illustrated in **Figure 3** (see also **Supplementary Movies 1**–**3**). More rapid movements of short telomeres were also observed with human cells (41). Together these observations suggest that a minimum number of telomeric repeats at chromosome ends could be required to anchor telomeres in heterochromatic areas of the nucleus. Such a location is expected to suppress transcription as well as activation of DNA damage signaling pathways.

Liquid-liquid phase transitions are known to be important for the regulation of transcription and DNA repair (42, 43) and have also been implicated in the function of telomeres (44). Unfortunately, studies of the role of liquid-liquid phase separation in telomere function (and biology in general) are complicated by the difficulty to reconstitute relevant phase transitions in the laboratory. It is possible that the transition between duplex telomere repeats and G4 DNA also acts as a switch, perhaps facilitating liquid-liquid phase transitions and/or activating DNA damage signaling pathways (45, 46). Other switch events at telomeres that need further study include the increased mobility of histones in telomere chromatin (15), the switch from RPA to CST during telomere replication (reviewed in (7), the initiation and suppression of telomere transcription (reviewed in (47) and the role of subtelomeric DNA and associated proteins including boundary elements such as CTCF in subtelomeric chromatin and telomere function (reviewed in (48). Further studies of these factors and their interplay promise to yield interesting results.

## Regulation of Telomere Length in Gametes and Early Embryos

The starting telomere length at the onset of life is presumably the telomere length present in the gametes from the parents. Little is known about the regulation of telomere length in oocytes of females throughout their reproductive lifespan. Perhaps all oocyte precursors express telomerase during embryonic development similar to spermatogonial stem cells in the mouse (49, 50). However, the average telomere length in human oocytes was reported to be around 1 kb shorter than in blastocysts, possibly reflecting loss of telomeric DNA during cell divisions of oocyte precursors *in utero* (51). Various factors implicated in regulating telomere length in embryos were recently reviewed (52). In males, the telomere length in sperm appears to slightly increase with age (53) and paternal age was found to correlate with telomere length in offspring (54). The most important events to shape the telomere length at birth and throughout life probably occur in the first few cell divisions of the fertilized oocyte when chromatin is completely reorganized (reviewed in (55)). First the dense chromatin in gametes is decompacted (56). The two-cell stage is characterized by demethylation and a unique gene expression profile that includes high expression of Zscan4 (57, 58). At this stage telomeres recombine, presumably to allow elongation and rescue of very short telomeres or damaged telomeres by break-induced replication (58, 59). Perhaps the telomere length in sperm chromosomes is the most important factor in adjusting the telomere length at the 2-cell stage. Suppression of G4 structures at telomeres could be particularly important when telomere chromatin is decompacted and telomerase is expressed at high levels. Undifferentiated human embryonic stem cells were reported to express high levels of G4 DNA (60). Telomere length adjustments at this stage could reflect a balance between single stranded G-rich DNA synthesized by telomerase, the CST proteins binding to such DNA, G4 DNA formation, G4 unwinding by helicase activity, C-strand “fill in” synthesis and various recombination processes. A major role for G4 DNA in the regulation of telomere length is supported by studies of mice. Laboratory mice*, Mus musculus*, with very long telomeres (>40 Kb) differ from *Mus spretus* mice with relatively short telomeres (~10 Kb) in RTEL1, the Regulator of Telomere Length helicase predicted to unwind guanine G4 structures at telomeres (29, 36, 61). More studies are needed to define the role of telomerase expression (62), primer sequestration, CST proteins and fill-in replication (63, 64) in the regulation of telomere length. Such studies should also clarify the role of factors suppressing the formation of G4 DNA at telomeres such as the CST complex (7), RAP1 (65) and POT1 (33) as well as helicases that can unwind G4 DNA structures that include, next to RTEL1, FANCJ (66) and BLM (67, 68). The protection of chromosome ends from DNA damage response pathways in early embryonic cells is very different from that in somatic cells (59, 69). How differences in end protection relate to telomere length adjustments will require further study. Such studies are complicated by the small numbers of relevant cells as well as the highly dynamic events within and between cell divisions during early embryogenic development.

Following telomere length adjustments at the 2-cell stage, telomerase expression in embryos may result in further elongation of telomeres. While the level of telomerase RNA appears to be the main limiting factor for telomerase activity in embryonic stem cells (70), it is possible that telomerase levels in blastocysts are further upregulated by low oxygen levels perhaps *via* induction of hTERT expression by the transcription factor hypoxia inducible factor 1 (HIF-1) alpha (71). The relatively high oxygen levels in typical tissue culture experiments (20%) do not match the much lower oxygen levels encountered by most cells *in vivo* and reducing oxygen levels during tissue culture increases the replicative potential of many cells *in vitro* (reviewed in (72). Culture of embryos at reduced oxygen levels could not only increase telomerase activity but also limit oxidative damage to telomeric DNA. Culture of embryos for assisted reproductive technologies should explore the effect of oxygen levels with some urgency given that reductions in telomere length prior to embryo implantation could have biological effects that only appear many years later. Future studies should also explore whether sex differences in telomere length and lifespan indeed reflect higher levels of dyskerin and telomerase in embryonic cells as was recently proposed (73). More generally, apart from overexpressing telomerase in cells, which seems neither feasible or advisable *in vivo*, measures that reduce exogenous damage to telomeric DNA represent perhaps the most effective ways to increase the replicative potential of cells *in vitro* and *in vivo*.

## Telomere Regulation *In Utero*

Telomerase levels are down-regulated in most human cells at some point during *in utero* growth and differentiation to limit the number of subsequent cell divisions. Both alternative splicing and transcriptional regulation of the telomerase transcriptase gene (*hTERT*) have been implicated in the silencing of telomerase activity (8, 74). However, details of the timing and degree of telomerase silencing during development and differences between specific cell types remain to be clarified. Downregulation of telomerase activity in somatic cells of long-lived animals was proposed to increase reproductive fitness by suppressing the growth of malignant tumors before reproduction (5). Several findings support this theory. Surprisingly, somatic mutations in dividing and non-dividing human tissues accumulate at a very similar rate of between 10-40 mutations per cell per year (75). In this study, cells of the germline were found to acquire only 1-2 mutations per year. Given that tumors arise by acquisition of mutations, one can wonder why DNA repair in somatic cells is not as effective as in cells of the germline. Apart from evolutionary arguments about the importance of germline versus somatic DNA in cells, the answer could involve a trade-off between the energy required to limit the acquisition of somatic mutations by improving DNA repair and other ways to suppress malignant growth prior to reproduction. Targeting telomerase expression levels in somatic cells to suppress tumors before reproduction could have provided a selective advantage that allowed lifespan to increase during evolution (5). Subsequent cell-type specific adjustments of telomerase levels could have allowed further increases in fitness. However, a requirement for tumor suppression by telomere shortening is that senescence or apoptosis is indeed enforced when telomeres become critically short. Given that most malignant tumors are deficient in p53, the Achilles heel of tumor suppression *via* telomere erosion in humans is perhaps loss of p53, resulting in failure to impose cell cycle arrest or apoptosis when telomeres are too short (6, 8, 76). Mechanistically, it seems possible that frequent loss of p53 on chr17p is related to the very short telomeres on that chromosome arm (77, 78). It is also tempting to speculate that multiple copies of p53 genes in long-lived animals such as elephants (79) reflect selection of more effective tumor suppression *via* DNA damage signals originating from short telomeres.

## Variable Telomerase Suppression in Somatic Cells

Next to the gradual loss of telomeric DNA with each cell division, sporadic loss of telomeric DNA also contributes to telomere shortening. Indeed, such losses are likely to underpin the strikingly heterogenous telomere length in human and murine cells. Sporadic truncation of telomeric DNA at specific chromosome ends was well-documented in cultured human fibroblasts (80). It is possible that the function of residual telomerase activity in most human somatic cells is not to prevent overall telomere shortening but to prevent premature senescence triggered by the sporadic loss of telomeric DNA (5). The need for such “telomere repair” by telomerase is expected to increase not only as a function of the overall telomere length but also as a function of the number of telomeres with damaged DNA. Differences between cells in the efficiency of telomere repair by telomerase argue against telomere shortening acting as a simple “mitotic clock” in all human somatic cells given that the number of cell divisions prior to replicative senescence could vary as a function of telomerase levels. Fibroblasts, with little if any telomerase activity, could encounter critical loss of telomeres and replicative senescence earlier than hematopoietic stem cells or T lymphocytes which have easily detectable telomerase activity (5). Telomerase activity could be regulated at many levels, ranging from regulation of gene expression and alternative splicing to variable processing of transcripts, proteins and assembly of the telomerase holoenzyme. Perhaps germinal center (GC) B cells represent the most extreme example of high telomerase activity in human somatic cells (81, 82). High levels of telomerase in GC B cells elongate telomeres relative to naive B cells, perhaps allowing some B cells to avoid the Hayflick limit altogether. Elongation of telomeres in GC B cells could reflect the need to enable more numerous replication cycles to support effective antibody production following clonal selection and affinity maturation. More effective antibody responses may have provided a selective advantage during evolution be it at the cost of increasing the risk of malignant transformation (5). This notion is compatible with observations indicating that B cell lymphomas in human adults are much more common than T cell lymphomas and that haploinsufficiency for telomerase causes pronounced defects in B cell responses (83). More generally, telomere-mediated limits in the proliferation of lymphocytes are likely to eventually compromise all different types of immune responses including those against other viruses and microorganisms (84) as well as tumor cells that can be recognized by the immune system (5). Of note, short telomere length in leukocytes was reported to correlate with poor outcome of COVID-19 infection irrespective of age (85).

## Loss of Telomere Repeats in Adult Human Cells

The decline in telomere length over a human lifetime has been most clearly documented for leukocyte subsets using fluorescence *in situ* hybridization and flow cytometry or “flow FISH” (86). Rapid decline in telomere length is observed in the first few years of life in line with rapid cell divisions as well as a “mitotic clock” ticking in hematopoietic stem cells (87, 88). These findings were recently discussed in the context of the “Telomere Erosion in Disposable Soma” theory of human aging (5). The essence of this theory is that replicative senescence could be advantageous as a tumor suppressor mechanism early in life but disadvantageous late in life by limiting cell proliferation and tissue regeneration. Interestingly, differences in average telomere length and lifespan between males and females are correlated and perhaps reflect differences in embryonic telomerase levels (73). Given the role of oxidative damage to telomeric DNA in telomere shortening (89), further studies of the effect of oxidative stress on leukocyte telomere length dynamics are of interest. Paradoxically, chronic and acute oxidative stress appear to have opposite effects on telomere length *in vivo*. Exposure to high oxygen levels in deep sea divers was shown to result in transient elongation of telomeres in leukocytes (90). In this study the telomere length returned to below baseline levels after a year. Perhaps replacement of cells lost by damage from oxidative stress increased the turnover of primitive stem cells (with longer telomeres) which would otherwise have stayed dormant longer. It is possible that a similar “robbing Peter to pay Paul” principle applies to the reported elongation of leukocyte telomeres during space flight in pure oxygen (91). In general, further long-term studies of telomere length in relation to health outcomes are needed to develop a clearer picture of telomere-mediated senescence and regeneration defects in various diseases that are more prevalent in the elderly.

## Conclusions

While much progress has been made in understanding various factors and pathways that play a role in regulating telomere length in different cell types many questions remain unsolved. Major variables are the length of telomeres in gametes and the processes at telomeres that occur between fertilization and embryo implantation. Variable suppression of telomerase levels in human somatic cells, ranging from little if any activity in fibroblasts to high levels in germinal center B cells, further complicate the picture. Whereas telomere shortening limits the proliferation of premalignant cells, it also limits immune responses. Variable suppression of telomerase in different cell types such as B and T lymphocytes could reflect ongoing selective forces. A major problem for the field is that all current telomere length measurements suffer from limitations (5, 92). Measurements of the average telomere length typically ignore variation in the length of telomere repeats at individual chromosomes which could be relevant for biological outcomes. Most techniques require thousands of cells and obscure differences between cells. Ideally, novel telomere length measurements should generate information about the average length as well as the distribution of telomere length in single cells. Single cell techniques are also needed to clarify the highly dynamic processes that result in adjustment of telomere length in the first few cell divisions after fertilization. With better telomere length measurements and more insight in telomere length regulation during development many of the current outstanding questions will be answered.

## Author Contributions

The author confirms being the sole contributor of this work and has approved it for publication.

## Funding

Work in the Lansdorp laboratory is funded by a Program Project Grant (#1074) from the Terry Fox Research Institute, a Project Grant (#PJT-159787) from the Canadian Institutes of Health Research, and a grant (#40044) from the Canadian Foundation for Innovation and the Government of British Columbia.

## Conflict of Interest

The author is a founder and shareholder of Repeat Diagnostics Inc., a company specializing in clinical telomere length measurements.

## Publisher’s Note

All claims expressed in this article are solely those of the authors and do not necessarily represent those of their affiliated organizations, or those of the publisher, the editors and the reviewers. Any product that may be evaluated in this article, or claim that may be made by its manufacturer, is not guaranteed or endorsed by the publisher.
