# Supplementary material for: Telomere Length Regulation
Source: Front Oncol. 2022 Jul 4;12:943622. doi: 10.3389/fonc.2022.943622 (PMC9289283; doi:10.3389/fonc.2022.943622)
Supplement: Supplementary file 4 [file DataSheet_1.pdf]

Supplementary Information

Time-lapse fluorescence microscopy and Supplementary Movies 1 – 3

For experimental details see (Lisaingo et al., 2014) and http://hdl.handle.net/2429/43056. Mouse embryonic stem (ES) cells tagged with fluorescent protein (Venus)-TRF1 were grown on coverglasses coated with gelatin. Time-lapse microscopy was performed using a Deltavision RT system (Applied Precision) with an Olympus IX inverted microscope and a CoolSnap HQ CCD camera. Cells were maintained in an environmental chamber at 37°C with 5% CO2 perfusion. Images were analyzed using SoftWoRx Suite software (Applied Precision) and Volocity (Improvision, Perkin Elmer). Exposure was 0.2 seconds and neutral density filters (transmission 10%) were used to minimize photobleaching effects.

Supplementary Movie 1. Telomere length dependent movement of telomeres in a single mouse embryonic stem cells expressing Venus TRF1. Short telomeres with faint Venus-TRF1 staining move around more rapidly than long telomeres brightly labeled with Venus-TRF1.

Supplementary Movie 2. Differences in the movement of short and long telomeres are more readily appreciated by zooming in on selected telomeres. Note the rapid movement of low intensity telomeres compared top high intensity telomere

Supplementary Movie 3. Example of image analysis used to measure the travel distance of individual telomeres in mouse ES cells expressing Venus-TRF1 shown in Figure 3. Note that the resolution after deconvolution in the Z direction is much less than in the X-Y plane explaining why some telomeres appear in multiple focal plans in Figure 2.

LISAINGO, K., URINGA, E. J. & LANSDORP, P. M. 2014. Resolution of telomere associations by TRF1 cleavage in mouse embryonic stem cells. *Mol Biol Cell,* 25**,** 1958-68.
